# Surveillance of antimicrobial resistance, phenotypic, and genotypic patterns of *Salmonella enterica* isolated from animal feedstuffs: Annual study

**DOI:** 10.14202/vetworld.2023.939-945

**Published:** 2023-05-07

**Authors:** Arsooth Sanguankiat, Nayika Pinniam, Phitsanu Tulayakul

**Affiliations:** Department of Veterinary Public Health, Faculty of Veterinary Medicine, Kasetsart University, Kamphaeng Saen, Nakhon Pathom. 73140, Thailand

**Keywords:** commercial feed compounds, genotype, *Salmonella* spp, serotype

## Abstract

**Background and Aim:**

Salmonellosis is a significant foodborne disease that causes serious illness in the gastrointestinal of humans and it is a public health problem worldwide. This study aimed to determine *Salmonella* spp. in animal feeds, its characteristic, serovar identification, genotyping, and drug sensitivity.

**Materials and Methods:**

*Salmonella* spp. from animal feedstuffs was collected from January 1 to December 31, 2017, with 657 samples. Serogroup classification was performed by using the slide agglutination test. Then, the samples were analyzed for genotype patterns using pulsed-field gel electrophoresis (PFGE) for DNA fingerprint and antibiotic sensitivity by Vitek^®^ 2 techniques.

**Results:**

A total of 80 samples (12.17 %) were found to be *Salmonella* positive; commercial feed compounds of 60 samples (75%); soybean meal of 10 samples (12.5%); pork meal of 5 samples (6.25%); a fish meal of three samples (3.75%) and poultry meal of 2 samples (2.5%). Serogroups B, C, D, and E were found in this study; 8 samples (10%), 39 samples (48.75%), 8 samples (10%), and 13 samples (16.25%), respectively. A total of 12 samples (15%) were not determined by serogrouping. The most common serovars were *Salmonella* Rissen (10%), *Salmonella* Mbandaka (8.75%), and *Salmonella* Livingstone (6.25%), which belong to serogroup C. Nine of eleven pulsotypes were detected when analyzed by PFGE, showed similarity index between 40.8 and 100 %. Antimicrobial susceptibility tests by Vitek^®^ 2 compact for 11 strains were classified into three groups: resistance to 4, 8, and 11 antibiotics, out of 20 antibiotics.

**Conclusion:**

This study revealed annual variation of *Salmonella* spp. Serovar, genotype, and phenotype from commercial feed compounds and raw materials of which involved people must be aware.

## Introduction

Salmonellosis is a significant foodborne disease categorized as a contagious disease in livestock according to the outbreak disease law in Thailand [1]. The condition causes serious illness in the gastrointestinal of humans, and it is a public health problem worldwide, including in Thailand. The bacteria are in Genus *Salmonella* and causes disease as a zoonosis, especially in livestock such as pigs, ruminants, and poultry [2–4]. *Salmonella* could be contaminated through any animal production process, especially in animal feeds, animal care, or animal product processes in the factory, and form Biofilm in eggshells [5–7]. Moreover, *Salmonella* can be found through the Animal gastrointestinal tract, and the infected animal sometimes shows no clinical sign of illness; however, it can be transmitted to other animal species [8]. Many studies of *Salmonella* spp. in various animals, including broiler chicken. It had been found that the infected *Salmonella enterica* broilers showed no clinical sign of disease, but the disease could be dispersed through their feces and the contaminated carcasses [9, 10].

In Thailand, the prevalence of *Salmonella* contamination in broiler standard farms was reported at 53.99% [11]. In Khon Kaen province, pigs, pig carcasses, water uses on farm, and worker in animal slaughterhouse of 27.14%, 36.67%, 19.51%, and 10.71%, respectively [12]. There was reported *Salmonella* contamination in animal feeds abroad, which contaminated 305/2622 samples (11.63%) with various serotypes detection of 78 serotypes [13]. The database collected from the Feed and Contaminants Program provided by USFDA reported that *Salmonella* spp. more than 25 serotypes could be frequently detected in feeds, especially *Salmonella* Senftenberg, *Salmonella* Montevideo, *Salmonella* Mbandaka, *Salmonella* Tennessee, *S. Typhimurium*, and so forth [14]. The contamination in animal feeds could be found in complete feed mills, which carried on through the heat–treated processes [15], of which the resource from cereals, beans containing high fat and high protein source of fishmeal [16]. The post-operative contamination of *Salmonella* in the finished product may come from the storage process, such as the cleanliness of the warehouse and pest control problems in the storage house [16]. Moreover, long-term *Salmonella* contamination has been reported in Animal feeds resource and complete animal feed [17]. Regarding genotyping, a molecular study of *Salmonella* samples collected in animals feed detected from four different big-named factories in Brazil. In the genotypic analysis using pulse-filed gel electrophoresis (PGFE) of 63 *Salmonella* samples out of 1269 samples, only six serovars were detected, such as Agona, Infantis, Montevideo, Orion, Senftenberg, and Worthington [18].

The objectives of this study were to study *Salmonella* spp. detected in animal feeds and its characteristic, serovar identification, genotyping, and determining drug sensitivity and gathered source database for further study.

## Materials and Methods

### Ethical approval

Ethical approval was not necessary for this study.

### Study period and location

This study was conducted from January 1^st^ to December 31^st^, 2017 at Kamphaeng Saen Veterinary Diagnostic Center, Faculty of Veterinary Medicine, Kasetsart University.

### Salmonella spp. determination

The determination of *Salmonella* spp. in this study used animal feeds collected from the samples sent to Kamphaeng Saen Veterinary Diagnostic Center, Faculty of Veterinary Medicine. The samples were then isolated for *Salmonella* spp. by culture in Macconkey agar and then randomly picked up one colony to culture in nutrient agar by incubation at 37°C for 24 h. After transfer, the target bacteria to culture on xylose lysine deoxycholate agar and brilliant green phenol red lactose sucrose agar, then did the biochemical test and transferred to store in skim milk and sent for serogrouping and genotyping analysis.

### Serogrouping and serotyping

The sample target bacteria were cultured with *Salmonella* O Polyvalent, Vi Antisera (S&A Reagent’s lab Ltd., Part, Bangkok, Thailand). Then drop 0.85% NaCl saline on a glass slide, pick up the target bacteria, then spread and mix them well with saline after dropping *Salmonella* OMA, OMB, OMC, OMD, and OME antisera on the sample and observing the reaction mixture’s precipitation. If testing with OMA antisera, continue testing with A, B, D, E, O: 21(L) antisera; if testing with OMB antisera, continue testing with C, F, G, H antisera. If testing with OMC antisera, then continue test with I, O: 17, 18, 28, 30, 35, 38 antisera, and if testing with OMD antisera, then continue test with O: 39, 40, 41, 42, 43, 44, 45 antisera. Finally, if testing with OME antisera, then continuing testing with O: 47, 48, 50, 51, 52, 53, 61 antisera. The serogroup-positive bacteria were isolated and then sent for serovar evaluation at S & A Reagent’s Lab. Ltd., Bangkok, Thailand.

### Pulsed-field gel electrophoresis

The pulsed-field gel electrophoresis (PFGE) technic has referred to the PulseNet Protocol for the molecular subtyping of *Salmonella* spp. (PulseNet, Centers for Disease Control and Prevention, Atlanta, Ga.) [19]. Selecting of *Salmonella* group C by the culture of the *Salmonella* in the skim milk on the XD agar and incubation at 37°C for 18–24 h., then pick up a single colony in the first step and transferred to the TSA agar and incubation at 37°C for 18–24 h. Pick up targeted bacteria and put in Cell suspension buffer (100 mM Tris 100:100 mM ethylenediaminetetraacetic acid [EDTA], pH 8.0) and adjust the optical density of Cell suspension into the McFarland of 8.0–10.0 using Den-1 MacFarland Densitometer (Grant-bio, Cambridge, UK). Then, Proteinase K enzyme 10 μL in 1.0% SeaKem Gold Agarose in TE buffer (10 mM Tris: 1 mM EDTA, pH 8.0) to generate the clot. After bringing the clot sample into Cell lysis buffer (50 mM Tris: 50mM EDTA, pH 8.0 + 1% Sarcosyl) volume of 5 mL in the centrifuge tube and added 25 μL of Proteinase K and gently mixed in 55°C water bath for 2 h. Wash the clotting plug twice with normal saline for 15 min/time, wash with Tris-EDTA buffer four times (15 min/time), and cut it for 2 mm thickness. For the DNA cutting, it starts with pre-heat with 100 μL restriction buffer at 37°C for 10 min, then added 200 μL restriction enzyme X*bal* at 37°C for 2 h and put in 25 μL of 0.5X Tris-borate EDTA buffer (TBE) (3150 mL: 10× TBE 150 mL, 3000 mL distilled water) at 25°C, 5 min. After that, put the plug on Comb and drop with Agarose gel (Seakem gold, Lonza, Maine, USA: 1.5 g, 0.5× TBE 150 mL) and then mold up with Agarose gel for 20 min until well settled. Bring the gel block into CHEF-MAPPER (Bio-Rad Laboratories, Richmond, USA), Set to program 1 (Runtime 19 h, Initial switch time 2.16 s, Final switch time 63.08 s, Voltage gradient 6V/cm, Ramping Linear, Start Initial milliamps 172, End Initial milliamps 185, Angle 60) then bring the gel to rinsed with RedSafe DNA staining solution, then take a picture under Ultraviolet with Gel Doc XR (Bio-Rad Laboratories) and analyze with Bionumeric V. 70,(Mérieux SA, biomerieux.com).

### Antibiotic sensitivity testing

The analysis used PFGE to group similar bacterial patterns. Then, the bacteria were sent for an antibiotic sensitivity test using a Vitek 2 compact (Biomerieux, Inc., North Carolina, USA). The Card type AST-GN65 card type and *Salmonella* spp. were selected, and the antibiotic sensitivity test with *Salmonella* spp. was as follows: ampicillin (AM), ampicillin plus amoxicillin (AM plus A), amoxicillin plus clavulanic acid (AMC), piperacillin (PIP), cefalexin (CN), cefovecin (CFO), cefpodoxime (CPO), ceftiofur (CFT), amikacin (AN), gentamicin (GM), tobramycin (TM), imipenem (IPM), trimethoprim-sulfamethoxazole (SXT), chloramphenicol (C), enrofloxacin (ENR), marbofloxacin (MRB), tetracycline (TE), tetracycline plus doxycycline (TE plus D), tetracycline plus minocycline and FT (TE plus MN), and nitrofurantoin.

## Results

**Figure-1:**
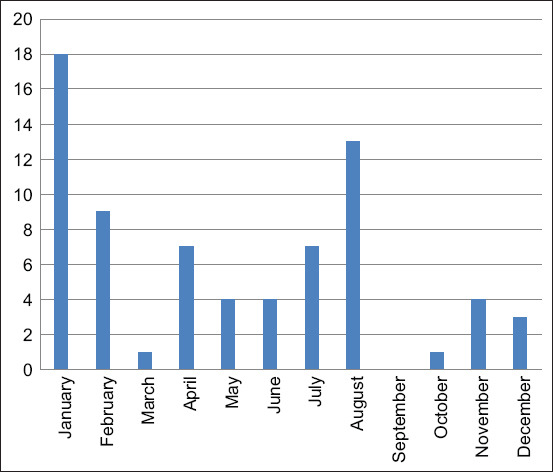
Number of annual feed samples sent to the Diagnostic Center in the year 2017.

**Figure-2:**
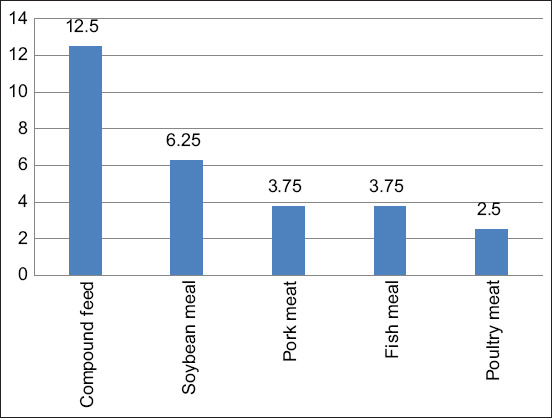
Positive *Salmonella* spp. (n = 60) detection from samples sent for diagnosis.

The analysis of the *Salmonella* spp. from 657 feed samples from January 1 to December 31, 2017, is shown in Figure-1. *Salmonella* spp. could be detected for 80/657 samples (12.17%) which could be categorized into 60/80 (75%) of animal feed samples, 10/80 (12.5%) in soybean meal samples, 5/80 (6.25%) in pork mill samples, 3/80 (3.75%) fish mill samples and 2/80 (2.5%) chicken meat samples as shown in Figure-2.

**Figure-3:**
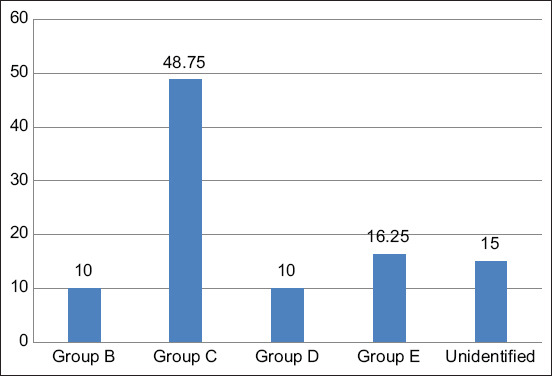
Serogroup identification according to Kauffmann-White Scheme using serogrouping of *Salmonella* O Polyvalent, Vi Antisera (S&A Reagents Lab Ltd., Part, Bangkok, Thailand), n = 60.

When operating the serogroup isolation by the slide agglutination technic according to the Kauffmann-White scheme using *Salmonella* O Polyvalent, Vi Antisera (S & A Reagents Lab. Ltd., Bangkok, Thailand). It was found that the *Salmonella* isolation was categorized for 35 samples (43.75%) in the OMA group, 41 samples (51.25%) in the OMB group, three samples (3.75%) in the OMC group, and 1 sample (1.25%) in the OME group. Then, all the samples were sent to identify the *Salmonella* antisera; it was found that 39 samples (48.75%) were in Group C, 13 samples (16.25%) group were in E, 8 samples (10%) were in Group D, 8 pieces (10%) were in Group B, and 12 samples (15%) were in the unidentified group as shown in Figure-3.

**Figure-4:**
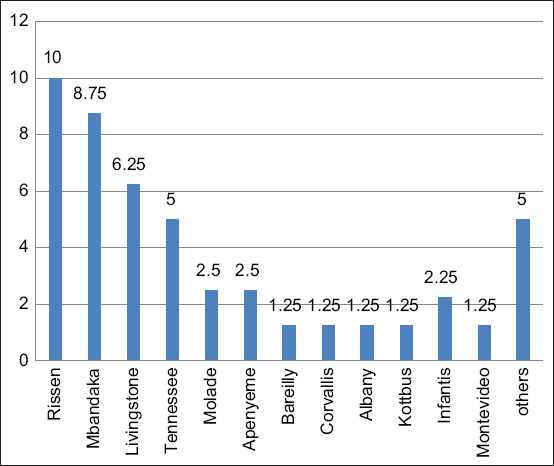
*Salmonella* Serovar isolation from serogroup C (n = 39) by S&A Reagent’s Lab Ltd., Part, Bangkok, Thailand.

The determination of serovar testing according to S & A Reagents Lab Ltd., found that 39 samples (48.75%) of the C group consisted of 8 samples (10%) of Serova *Salmonella* Rissen, seven samples (8.75%) of Mbandaka, five samples (6.25%) of *Salmonella* Livingstone, 4 samples (5%) of Tenessee, two samples (2.5%) of Bolade, two samples (2.5%) of Apenyeme, two samples (2.5%) of Bareilly 1 sample (1.25%) of Corvallis, 1 sample (1.25%) of Albany, one sample (1.25%) Kottbus, 1 sample (1.25%) of Infantis and 1 sample (1.25%) of Montevideo. Moreover, some isolation fell into other groups, such as Group B, which consisted of 2 samples (2.5%) of serovar Agona and Group C, which consisted of 1 sample (1.25%) of serovar *Lexington* and 1 sample (1.25%) of Senftenberg as shown in Figure-4.

**Figure-5:**
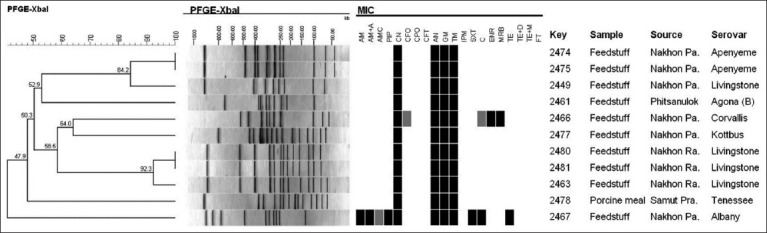
Dendrogram demonstrates of *Salmonella* pattern and Similarity index analysis by pulsed-field gel electrophoresis. AM=Ampicillin, AM+A=Ampicillin plus amoxicillin, AMC=Amoxicillin plus clavulanic acid, PIP=Piperacillin, CN=Cefalexin, CFO=Cefovecin, CPO=Cefpodoxime, CFT=Ceftiofur, AN=Amikacin, GM=Gentamicin, TM=Tobramycin, IPM=Imipenem, SXT=Trimethoprim-sulfamethoxazole, C=Chloramphenicol, ENR=Enrofloxacin, MRB=Marbofloxacin, TE=Tetracycline, TE+D=Tetracycline plus doxycycline, TE+M=Tetracycline plus minocycline and FT.

The study of PFGE was commenced by selecting 11 samples of *Salmonella* serogroup C 11 and determined according to the PulseNet Protocol for *Salmonella* spp. (PulseNet, Centers for Disease Control and Prevention) [19]. It was found that the appearance of DNA fingerprints could be categorized into nine pulsotypes where the similarity index was between 47.9 and 100 pulsotypes after analyzing with the Bionumeric program V.70, as shown in Figure-5.

**Figure-6:**
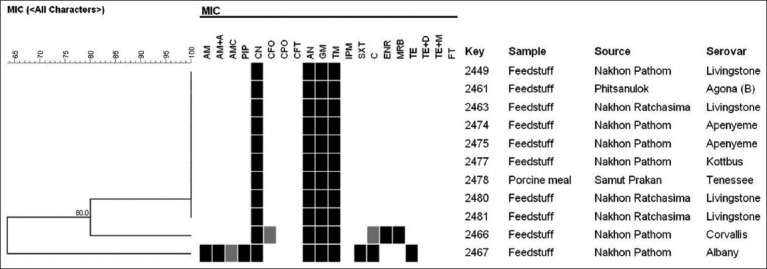
Dendrogram demonstrates of drug sensitivity pattern and similarity index by pulsed-field gel electrophoresis. AM=Ampicillin, AM+A=Ampicillin plus amoxicillin, AMC=Amoxicillin plus clavulanic acid, PIP=Piperacillin, CN=Cefalexin, CFO=Cefovecin, CPO=Cefpodoxime, CFT=Ceftiofur, AN=Amikacin, GM=Gentamicin, TM=Tobramycin, IPM=Imipenem, SXT=Trimethoprim-sulfamethoxazole, C=Chloramphenicol, ENR=Enrofloxacin, MRB=Marbofloxacin, TE=Tetracycline, TE+D=Tetracycline plus doxycycline, TE+M=Tetracycline plus minocycline and FT.

The antibiotic sensitivity testing of 11 selected *Salmonella* spp. found that only six antibiotic-sensitive items as CPO, CFT, IPM, TE plus D, TE plus MN, and nitrofurantoin. There also found the median sensitivity for AMC for 1/11 (9.09%), C for 1/11 (9.09%), and CFO for 1/11 (9.09%). Antibiotic resistance of 100% (11/11 samples) was found for tobramycin, CN, AN, and GM. Furthermore, a lower resistance of 9.09% (1/11 samples) was found for C, Amoxicillin, AM plus A, PIP, SXT, ENR, MRB, and TE. Moreover, after analyzing the antibiotic sensitivity using Bionumeric V.70, demonstrated three phenotypes group found in Group 1 were resistant to 4/20 antibiotics, Group 2 resistant to 8/20 antibiotics, and Group 3 resistant to 11/20 antibiotics, as shown in Figure-6.

## Discussion

The study isolated 80 samples (12.17%) to be *Salmonella* positive. This finding is in accordance with the report of *Salmonella* finding in animal feeds in the United States of America 2002–2009, where *Salmonella* spp. could be detected for 12.5% (257/2,058 samples) and reported to be most significant in the raw feed components in Australia [14, 20]. There were also reports of *Salmonella* spp. for 11.63% (305/2,622 samples) during 2007–2011 and 2008–2017 in the United States [13, 21]. Whereas the serogroup typing using the slide agglutination test according to *Salmonella* O Polyvalent, Vi Antisera (S & A Reagents Lab. Ltd.), it was found that 48.75 % (39/80 samples) of the positive to *Salmonella* were in serogroup C. Serogroup C is the most abundant group in analyzed animal feeds since this group is essential regarding animal and human health. It is mainly found in Asia and America [22]. Moreover, it was reported that *Salmonella* Infantis belonging to serogroup C, causes disease outbreaks according to Thailand’s Disease outbreak law 2015. Serova (53.8%) was common in slaughterhouses reported in Belgium [1, 23]. In terms of serovar testing, it was found that the most frequently detected was 10% (8/80 samples) of *S*. Rissen, *S*. Mbandaka, *S*. Livingstone, *S*. Tennessee for 8.75% (7/80 samples), 6.25% (5/80 samples) and 5% (4/80%), respectively, which are usually isolated in animal feeds [14]. They were reported to be found in chicken and pork products, primarily in the study of poultry meat in Northern Thailand. Among the 27 serovars detected, the serovar Covallis, Singapore, Kentucky, and Agona were most frequently detected. On the other hand, *Salmonella* Napoli and *Salmonella* Derby became most commonly isolated from humans in Italy [24–26]. Moreover, it was also found that *S*. Rissen (45.3%) is mainly found in many processes of pork production, which is in the same direction as the *S*. Rissen found in animal feeds which could be correlated with the contamination of *S*. Rissen in the pork production line. However, the scenario was quite different in the study of Australian feed mills [27]; during the monitoring of feed mills for 16 years, 23,963 samples were collected and determined in Australia. The serotype most frequently isolated from raw materials was *Salmonella* Agona, while *Salmonella* Anatum was most commonly isolated from the equipment and finished feed. *Salmonella* was widely found in commercial poultry farms in Nigeria and *S*. Kentucky and *S*. Isangi were the most prevalent serotypes [28].

After selecting 11 samples of serogroup C *Salmonella* to evaluate the molecular characteristic using pulsed-field get electrophoresis (PFGE) according to PulseNet Protocol of *Salmonella* spp. (PulseNet, Centers for Disease Control and Prevention) [19]. Nine pulsotype of DNA fingerprint characteristics were found, showing a similarity index between 47.9 and 100 for each pulsotype. From characteristic genotypic findings, it found a 100 % similarity of two pairs for 2474–2475 for serovar Apenyeme and 2480–2481 for serovar *S*. Livingstone. Interestingly, it could identify the serovar *S*. Livingstone into nine distinct characteristics, even if it came from the same bacteria. It also reported that *Salmonella* collected from different origins showed different genotypic patterns, of which the similarity index was between 50.3 and 92.3. The results were in accordance with the finding of the antibiotic sensitivity test on *S*. Rissen, that the phenotypic of antibiotic patterns differ from the genotype found, as reported by Hendriksen *et al*. [29], Herikstad *et al*. [30].

The results on the antibiotic test using Vitek® 2 compact (Biomerieux, Inc.) tested *Salmonella* were resistant to CN, AN, GM, and Tobramycin. Even though the first- and second-generations of Cephalosporin and Aminoglycoside could be used to kill *Salmonella* spp., they could not be reported as sensitive drugs for *Salmonella* spp. [31] because it has never been used in an actual farm situation except in a report of beta-lactamase, aminoglycoside, and sulphonamide resistance genes detected in poultry feeds from Kenya [32]. Moreover, two serovars of serovar *Corvalis* were found to have intermediate sensitivity to CFO and C but resistance to ENR and MRB. At the same time, the serovar Albany sample was intermediately sensitive to amoxicillin plus clavulanic acid and resistant to PIP, AM, AM plus A, SXT, C, and TE. The antibiotic sensitivity found in this study was similar to the finding of *Salmonella* cultured in animal feeds in Poland, which found antibiotic resistance of 5.6% to C, 22.2 % to TE, and 5.6% to β-lactam [33]. Moreover, another antibiotic resistance was reported in *Salmonella* isolated in chicken meat. Therefore, the most resistant was nalidixic acid (31%), then AM (24%), TE (19%), and Sulfamethoxazole-Trimethoprim (8%) [34]. In addition, it was reported that *Salmonella* isolated recovered from Australian feed mills found that 11% (48/453 samples) were resistant to two or more antimicrobials, whereas 79% (356/453 samples) were still susceptible to the antimicrobial from the study [29]. Therefore, it was able to group different phenotypic according to antibiotic sensitivity into Group 1 resistance of 4/20 drugs, Group 2 resistance of 8/20 drugs, and Group 3 resistance of 11/20 drugs. However, there was a report on whole genome sequences of four multidrug resistance of *Salmonella* spp. they were isolated from poultry in Brazil. They identified that the IncHI2-HT2 megaplasmid carried a resistome containing eleven resistance genes and four heavy metals resistance operons [35]. The phenotypic characteristic depends on the bacteria’s resistance to genotypic patterns. Moreover, it is also related to the antibiotic resistance mechanism by integrons and plasmid transfer of resistance gene of which *Salmonella* spp. could be adopted by the resistance gene to a different bacterial group and the same *Salmonella* spp. group [34, 36].

## Conclusion

The study of genotypic and phenotypic characteristics of *Salmonella* spp. in animal feeds showed distinctive characteristics, even having similar serovar. Pulse-field gel electrophoresis technic helps separate various *Salmonella* spp. in the same serovar group. The antibiotic sensitivity test reveals that similar genotypic *Salmonella* has similar antibiotic resistance patterns. Although the antibiotic resistance in *Salmonella* may not directly affect human health, it could affect animals through production processes from contamination in animal feedstuffs and indirectly human consumer. The database linkage between human health and animal products related to the *Salmonella* problem was limited. It will be improved to show the future relationship between both health sectors. Therefore, the surveillance of bacterial sources and control measures was essential and needed to be focused on to alleviate the contamination of significant bacteria, especially *Salmonella* spp., in food chains for food safety concerns.

## Authors’ Contributions

AS: Study design, sample collection, statistical analysis, and drafted the manuscript. NP: Sample collection and analysis and phenotypic and genotypic characterization. PT: Study design and drafted and revised the manuscript. All authors have read, reviewed, and approved the final manuscript.

## References

[1] Animal Disease Outbreak Law (2015). Bureau of Animal Disease Prevention and Control. Vol. 132., Ch. 14A.

[2] De Knegt LV, Pires S, Hald T (2015). Using surveillance and monitoring data of different origins in a *Salmonella* source attribution model:A European Union example with challenges and proposed solutions. Epidemiol. Infect..

[3] Magossi G, Cernicchiaro N, Dritz S, Houser T, Woodworth J, Jones C, Trinetta V (2019). Evaluation of *Salmonella* presence in selected United States feed mills. Microbiologyopen.

[4] Kumar Y, Singh V, Kumar G, Gupta N.K, Tahlan A.K (2019). Serovar diversity of *Salmonella* among poultry. Indian J. Med. Res..

[5] Guillén S, Marcén M, Álvarez I, Mañas P, Cebrián G (2020). Stress resistance of emerging poultry-associated *Salmonella* serovars. Int. J. Food Microbiol..

[6] Waldman J, Souza M.N, Fonseca A.S.K, Ikuta N, Lunge V.R (2020). Direct detection of *Salmonella* from poultry samples by DNA isothermal amplification. Br. Poult. Sci..

[7] Silva P.L.A.P, Goulart L.R, Reis T.F.M, Mendonça E.P, Melo R.T, Penha V.A.S, Peres P.A.B.M, Hoepers P.G, Beletti M.E, Fonseca B.B (2019). Biofilm formation in different *Salmonella* Serotypes isolated from poultry. Curr. Microbiol..

[8] Bell C, Kyriakides A (2001). *Salmonella*:A Practical Approach to the Organism and its Control in Foods. Wiley-Blackwell, United States.

[9] Rigby C.E, Pettit J.R, Baker M.F, Bentley A.H, Salomons M, Lior H (1980). Flock infection and transport as sources of *Salmonellae* in broiler chickens and carcasses. Can. J. Comp. Med..

[10] Brooks L.A, Bailey M.A, Krehling J.T, Chasteen K.S, Macklin K.S (2021). A comparison of colonizing ability between *Salmonella* Enteritidis and *Salmonella* Heidelberg in broiler chickens challenged through feed administration. Foodborne Pathog. Dis..

[11] Suddee W (2014). Prevalence and risk factors of *Salmonella* spp. In:Standard Broiler Farms in Year 2014. Bureau of Animal Disease Prevention and Control. Department of Livestock Development, Bangkok.

[12] Angkititrakul S, Sitthikol D (2014). Prevalence of *Salmonella* spp. Isolated from pork, pig carcasses and used water and worker in a slaughterhouse in Khonkaen province. Khonkaen Vet. J..

[13] Hsieh Y.C, Poole T.L, Runyon M, Hume M, Herrman T.J (2016). Prevalence of nontyphoidal *Salmonella* and *Salmonella* strains with conjugative antimicrobial-resistant serovars contaminating animal feed in Texas. J. Food Prot..

[14] Li X, Bethune L.A, Jia Y, Lovell R.A, Proescholdt T.A, Benz S.A, McChesney D.G (2012). Surveillance of *Salmonella* prevalence in animal feeds and characterization of the *Salmonella* isolates by serotyping and antimicrobial susceptibility. Foodborne Pathog. Dis..

[15] Jones F.T, Richardson K.E (2004). *Salmonella* in commercially manufactured feeds. Poult. Sci..

[16] Hald T, Wingstrand A, Pires S.M, Vieira A, Domingues A.R, Lundsby K, Thrane C (2012). Assessment of the Human-Health Impact of *Salmonella* in Animal Feed. National Food Institute, Technical University of Denmark, Denmark.

[17] Jones F.T (2011). A review of practical *Salmonella* control measures in animal feed. J. Appl. Poult. Res..

[18] Pellegrini D.D.C, Paim D.S, De Lima G.J.M, Pissetti C, Kich J.D, de Itapema Cardoso M.R (2015). Distribution of *Salmonella* clonal groups in four Brazilian feed mills. Food Contol.

[19] CDC (2017). Standard Operating Procedure for PulseNet PFGE of *Escherichia coli* O157:H7, *Escherichia coli* Non-O157 (STEC), *Salmonella* Serotypes, *Shigella sonnei* and *Shigella flexneri*. PNL05. CDC, United States.

[20] Parker E.M, Valcanis M, Edwards L.J, Andersson P, Mollenkopf D.F, Wittum T.E (2022). Antimicrobial-resistant *Salmonella* is detected more frequently in feed milling equipment than in raw feed components or processed animal feed. Aust. Vet. J..

[21] Yin X, M'ikanatha N.M, Nyirabahizi E, McDermott P.F, Tate H (2021). Antimicrobial resistance in non-Typhoidal *Salmonella* from retail poultry meat by antibiotic usage-related production claims-United States, 2008–2017. Int. J. Food Microbiol..

[22] Fuche F.J, Sow O, Simon R, Tennant S.M (2016). *Salmonella* serogroup C:Current status of vaccines and why they are needed. Clin. Vaccine Immunol.

[23] Zeng H, De Reu K, Gabriël S, Mattheus W, De Zutter L, Rasschaert G (2021). *Salmonella* prevalence and persistence in industrialized poultry slaughterhouses. Poult. Sci..

[24] Angkititrakul S, Chomvarin C, Chaita T, Kanistanon K, Waethewutajarn S (2005). Epidemiology of antimicrobial resistance in *Salmonella* isolated from pork, chicken meat and humans in Thailand. Southeast Asian J. Trop. Med. Public Health.

[25] Vidayanti I.N, Sukon P, Khaengair S, Pulsrikarn C, Angkittitrakul S (2021). Prevalence and antimicrobial resistance of *Salmonella* spp. Isolated from chicken meat in upper northern Thailand. Vet. Integr. Sci.

[26] Leati M, Zaccherini A, Ruocco L, D'Amato S, Busani L, Villa L, Barco L, Ricci A, Cibin V (2021). The challenging task to select *Salmonella* target serovars in poultry:The Italian point of view. Epidemiol. Infect..

[27] Parker E.M, Edwards L.J, Mollenkopf D.F, Ballash G.A, Wittum T.E, Parker A.J (2019). *Salmonella* monitoring programs in Australian feed mills:A retrospective analysis. Aust. Vet. J..

[28] Jibril A.H, Okeke I.N, Dalsgaard A, Kudirkiene E, Akinlabi O.C, Bello M.B, Olsen J.E (2020). Prevalence and risk factors of *Salmonella* in commercial poultry farms in Nigeria. PLoS One.

[29] Hendriksen R.S, Bangtrakulnonth A, Pulsrikarn C, Pornreongwong S, Hasman H, Song S. W, Aarestrup F.M (2008). Antimicrobial resistance and molecular epidemiology of *Salmonella* Rissenfrom animals, food products, and patients in Thailand and Denmark. Foodborne Pathog. Dis..

[30] Herikstad H, Motarjemi Y, Tauxe R.V (2002). *Salmonella* surveillance:A global survey of public health serotyping. Epidemiol. Infect.

[31] Clinical and Laboratory Standards Institute (CLSI) (2013) Performance Standard for Antimicrobial Susceptibility Testing;Twenty-Third Informational Supplement Clinical and Laboratory Standards Institute, United States.

[32] Ngai D.G, Nyamache A.K, Ombori O (2021). Prevalence and antimicrobial resistance profiles of *Salmonella* species and *Escherichia coli* isolates from poultry feeds in Ruiru Sub-County, Kenya. BMC Res. Notes.

[33] Wasyl D, Hoszowski A (2004). Antimicrobial resistance of *Salmonella* isolated from animals and feed In Poland. Bull. Vet. Inst. Pulawy.

[34] Chuanchuen R, Pathanasophon P, Khemtong S, Wannaprasat W, Padungtod P (2008). Susceptibilities to antimicrobials and disinfectants in *Salmonella* isolates obtained from poultry and swine in Thailand. J. Vet. Med. Sci..

[35] Galetti R, Filho R.A.C.P, Ferreira J.C, Varani A.M, Sazinas P, Jelsbak L, Darini A.L.C (2021). The plasmidome of multidrug-resistant emergent *Salmonella* serovars isolated from poultry. Infect. Genet. Evol..

[36] Alcaine S.D, Warnick L.D, Wiedmann M (2007). Antimicrobial resistance in nontyphoidal *Salmonella*. J. Food Prot..

